# Prognostic value of uPA and p53 accumulation measured by quantitative biochemical assays in 1245 primary breast cancer patients: a multicentre study

**DOI:** 10.1038/sj.bjc.6690389

**Published:** 1999-05-01

**Authors:** P Broët, F Spyratos, S Romain, V Quillien, A Daver, G Ricolleau, A Rallet, C Toulas, B Asselain

**Affiliations:** Département de Biostatistiques, Institut Curie, 26 rue d'Ulm, 75005, Paris, France; Centre René Huguenin, 35 rue Dailly, 92211 St-Cloud, France; CHRU, 80 Bd P Dramard, 13916 Marseille, France; Centre E Marquis, BP 6279, 35000 Rennes, France; Centre P Papin, 2 rue Moll, 49033 Angers, France; Centre R Gauduchea, Bd J Monod, 44805 St-Herblain, France; Centre J Godinot, 1 rue du Général Koenig, 51056 Reims, France; Centre C Regaud, 20 rue du Pont St-Pierre, 31052 Toulouse, France

**Keywords:** urokinase plasminogen activator, p53, luminometric immunoassay, survival analysis, breast cancer

## Abstract

The purpose of this retrospective multicentre study was to assess the prognostic value of urokinase plasminogen activator (uPA) and p53 levels in a large series of primary breast cancer, using an automatic quantitative luminometric method. Samples of 1245 operable breast tumours were collected from seven French institutions and patients were followed for a median of 75 months. The median uPA and p53 levels assayed in cytosols by means of the immunoluminometric technique (LIA) were 0.31 and 0.20 ng mg^−1^ of protein respectively. In univariate analysis, high levels of uPA and p53 were associated with shorter disease-specific survival, disease-free interval, and distant recurrence-free interval. The 5-year survival rates were 95.5% among patients with uPA values below the 20th percentile, and 77.5% in those with values above the 80th percentile. The 5-year survival rates were 91.0% in patients with p53 values below the 20th percentile, and 77.6% in those with values above the 80th percentile. In multivariate analysis, the risk of disease-related death increased with uPA levels after adjustment for tumour size, histological grade, lymph node involvement, and estrogen receptor status. A high level of uPA was also related to a shorter disease-free interval and distant recurrence-free interval. In node-negative patients, a high level of uPA remained strongly related to the three outcomes. When adjusted for other prognostic factors, p53 was no longer significantly related to the outcomes. Given its rapidity and simple application to routinely prepared cytosols, this LIA may be useful for evaluating the prognostic impact of uPA in primary breast cancer, particularly in node-negative patients. According to our results, the prognostic value of p53 accumulation is limited when uPA is included in multivariate analysis. © 1999 Cancer Research Campaign


*British Journal of Cancer* (1999) **80**(3/4), 536–545
© 1999 Cancer Research Campaign
Article no. bjoc.1998.0389

### Prognostic value of uPA and p53 accumulation measured by quantitative biochemical assays in 1245 primary breast cancer patients: a multicentre study

**P Broët[1], F Spyratos[2], S Romain[3], V Quillien[4], A Daver[5], G Ricolleau[6], A Rallet[7], C Toulas[8] and B Asselain[1]**

[1]Département de Biostatistiques, Institut Curie, 26 rue d'Ulm, 75005, Paris, France; [2]Centre René Huguenin, 35 rue Dailly, 92211 St-Cloud, France; [3]CHRU, 80 Bd P Dramard, 13916 Marseille, France; [4]Centre E Marquis, BP 6279, 35000 Rennes, France; [5]Centre P Papin, 2 rue Moll, 49033 Angers, France; [6]Centre R Gauducheau, Bd J Monod, 44805, St-Herblain, France; [7]Centre J Godinot, 1 rue du Général Koenig, 51056 Reims, France; [8]Centre C Regaud, 20 rue du Pont St-Pierre, 31052 Toulouse, France

**Summary** The purpose of this retrospective multicentre study was to assess the prognostic value of urokinase plasminogen activator (uPA) and p53 levels in a large series of primary breast cancer, using an automatic quantitative luminometric method. Samples of 1245 operable breast tumours were collected from seven French institutions and patients were followed for a median of 75 months. The median uPA and p53 levels assayed in cytosols by means of the immunoluminometric technique (LIA) were 0.31 and 0.20 ng $mg^{-1}$ of protein respectively. In univariate analysis, high levels of uPA and p53 were associated with shorter disease-specific survival, disease-free interval, and distant recurrence-free interval. The 5-year survival rates were 95.5% among patients with uPA values below the 20th percentile, and 77.5% in those with values above the 80th percentile. The 5-year survival rates were 91.0% in patients with p53 values below the 20th percentile, and 77.6% in those with values above the 80th percentile. In multivariate analysis, the risk of disease-related death increased with uPA levels after adjustment for tumour size, histological grade, lymph node involvement, and estrogen receptor status. A high level of uPA was also related to a shorter disease-free interval and distant recurrence-free interval. In node-negative patients, a high level of uPA remained strongly related to the three outcomes. When adjusted for other prognostic factors, p53 was no longer significantly related to the outcomes. Given its rapidity and simple application to routinely prepared cytosols, this LIA may be useful for evaluating the prognostic impact of uPA in primary breast cancer, particularly in node-negative patients. According to our results, the prognostic value of p53 accumulation is limited when uPA is included in multivariate analysis.

**Keywords:** urokinase plasminogen activator; p53; luminometric immunoassay; survival analysis; breast cancer

Besides the classical prognostic factors used in clinical practice, a series of biological markers have been tested for their predictive value regarding survival or treatment responses in breast cancer (Figueroa et al, 1993; Porter-Jordan and Lippman, 1994). Most interpretations of the data have been complicated by the use of different techniques for measurement and analysis, but such new parameters could be of interest in selecting high-risk groups, especially among node-negative patients. Among the plethora of potentially useful markers that have been identified, two appear to be of particular interest: urokinase-type plasminogen activator (uPA) and p53. UPA has a role in the destruction of the basement membrane and in invasion (Andreasen et al, 1997), and several studies from different countries indicate that uPA overexpression, measured by biochemical methods, is strongly related to shorter disease-free and overall survival, particularly in node-negative patients (Duffy, 1996). These results led a German group to initiate a clinical trial in node-negative patients (Jänicke et al, 1994*b*).

Mutations of the tumour suppressor gene p53 are the most frequent known genetic alterations in all human cancers. Most biologically significant mutations hinder the participation of p53 in maintaining genomic stability (Gottlieb and Oren, 1996). p53 gene alterations and accumulation of p53 protein detected mainly by immunohistochemistry are generally related to shorter disease-free survival (Velculescu and El-Deiry, 1996). Mis-sense mutations normally result in an increased half-life of the protein product and accumulation of mutant p53 protein in the nucleus. Methodological differences and variability in immunohistochemical assays may explain discordant results regarding the prognostic significance of p53 accumulation in breast cancer (Allred et al, 1993; Thor and Yandell, 1993).

In this study we tested an automatic quantitative assay for uPA and p53 protein accumulation, and evaluated the prognostic value of these markers in a multicentre study. The study included 1245 tumours from primary breast cancer patients from seven French institutions. Patients were followed for a median of 75 months. Measurements were made using an automatic quantitative biochemical method (Borg et al, 1995; De Witte et al, 1996; Fernö et al, 1996; Berns et al, 1997; Bouchet et al, 1998; Peyrat et al, 1998), which is feasible for routine determination on cytosols prepared for hormone receptor assays.

*Received 23 July 1998*
*Revised 12 November 1998*
*Accepted 25 November 1998*

*Correspondence to:* F Spyratos

The participating laboratories are members of the Groupe de Biopathologie Tissulaire et Moléculaire of the Fédération Nationale des Centres de Lutte Contre le Cancer (FNCLCC).

### MATERIALS AND METHODS

### Patients' characteristics

This study involved 1245 patients diagnosed and treated at seven cancer centres between the beginning of 1980 and the end of 1994. The patients presented with unilateral non-metastatic breast tumours and were initially treated with modified mastectomy or breast-conserving surgery. The following information was recorded for each patient: age at diagnosis, hormone status, clinical stage, histological classification and grade, pathological tumour size, axillary lymph node involvement, steroid receptor status, uPA and p53 values, and primary treatment.

The median age was 58 years (range 24–88). A total of 223 patients (18%) were under 45 years of age, 670 (54%) were between 46 and 65 years, and 352 (28%) were over 65 (Table 1).

#### *Menopausal status was defined as 2 years after complete arrest of periods*

Sixty-six per cent of the patients were post-menopausal. Significant differences ($P < 0.001$) in mean age was observed between the centres. According to UICC criteria (UICC, 1988), 13 (1%) patients had stage 0 disease, 239 (19%) stage I, 884 (72%) stage II and 99 (8%) stage III. The median pathologic tumour size was 22 mm (0–150). Most of the patients (89%) had invasive ductal carcinoma and 8% had lobular carcinoma; the other histological types were analysed as a group (3%). A total of 1141 tumours were graded according to the Scarff Bloom and Richardson (SBR) classification (Bloom and Richardson, 1957; Scarff and Torloni, 1968) (SBR I 16%, SBR II 58%, SBR III 26%). Axillary lymph node status (mean number examined 14) was assessed by histological examination in 1141 cases. Six hundred and sixteen patients (54%) were node-negative, 319 (28%) had one to three invaded nodes, and 206 (18%) had more than four invaded nodes. Significant differences in median tumour size, clinical stage, the proportion of positive or negative lymph nodes and histological grade were observed among the centres ($P < 0.0001$). The primary treatment was tumorectomy (63.5%) or mastectomy with axillary dissection (36.5%). Of the entire population, 84% had radiotherapy following surgery. Nineteen per cent of the patients received chemotherapy, 25% received hormone therapy and 14% received both.

### Tissue extracts

Tumour tissue was collected from 1245 patients treated in six French cancer centres and in the Assistance Publique of Marseille.

### Oestrogen (ER) and progesterone (PR) receptor assays

A section of tumour tissue was frozen in liquid nitrogen immediately after surgery. The hormone receptor status of the tumour was recorded at the time of surgery. The tumour was considered to be steroid receptor-positive if ER and PR values exceeded 10 fmol $mg^{-1}$ cytosol protein. Quality control was based on regular testing of both internal controls (pooled cytosols) and European Organization for Research and Treatment of Cancer (EORTC, 1980) controls. Approximately two-thirds of the tumours were positive (ER = 73%, PR = 64%). Significant differences in the proportions of ER and PR positivity were observed among the different centres ($P < 0.0001$).

**Table 1** Population characteristics

| Characteristics | Number of patients | (%) |
|---|---|---|
| Age | | |
| Mean (years) | 58 | |
| Range (min, max) | 24–88 | |
| ≤ 45 | 223 | (18) |
| 46–65 | 670 | (54) |
| > 65 | 352 | (28) |
| Hormonal status | | |
| Premenopausal | 414 | (34) |
| Post-menopausal | 813 | (66) |
| ND | 18 | |
| UICC stage | | |
| 0 | 13 | (1) |
| I | 239 | (19) |
| II | 884 | (72) |
| III | 99 | (8) |
| ND | 10 | |
| Pathological tumour size (pT) | | |
| ≤ 20 mm | 428 | (35) |
| 21–50 mm | 731 | (60) |
| > 50 mm | 55 | (5) |
| ND | 31 | |
| Histology | | |
| Ductal | 1105 | (89) |
| Lobular | 105 | (8) |
| Other histology | 35 | (3) |
| Histological grading (SBR) | | |
| I | 180 | (16) |
| II | 660 | (58) |
| III | 301 | (26) |
| ND | 104 | |
| Axillary lymph nodes (pN) | | |
| None | 616 | (54) |
| 1–3 | 319 | (28) |
| ≥ 4 | 206 | (18) |
| ND | 104 | |
| ER status | | |
| Negative | 333 | (27) |
| Positive | 905 | (73) |
| ND | 7 | |
| PR status | | |
| Negative | 447 | (36) |
| Positive | 790 | (64) |
| ND | 8 | |

ND: not determined, ER: estrogen receptor, PR: Progesterone receptor.

For uPA and p53 assays, four laboratories (laboratories 2, 4 and 5 in Table 2) used cytosols prepared for hormone receptor assays at the time of initial surgery and then frozen in liquid nitrogen. The other three laboratories prepared cytosols specifically for this study by homogenizing fragments of frozen tumours in the same hormone receptor buffer (10 mM Tris-HCl, pH =7.4, 1.5 mM EDTA, 10 mM $Na_2MoO_4$, 0.5 mM dithiotreitol, 10% glycerol). After centrifugation for 1 h at 105 000 ***g***, supernatants were aliquoted and stored in liquid nitrogen until analysis (maximum 3 months). uPA and p53 assays were performed in each centre with the same methodology. Internal controls (pooled cytosols) were used for each parameter in each series of tests. Cytosol extract protein concentrations were determined using the BCA assay (Pierce, Rockford, IL, USA). All assays were done in duplicate.

### Luminometric immunoassay for p53 and uPA

p53 ($n$ = 1231) and uPA ($n$ = 1094) were assayed by using a luminometric immunoassay (LIA-mat p53 and LIA-mat uPA, Byk

© Cancer Research Campaign 1999

**Table 2** uPA and p53 according to the participating centres

| Centre | uPA | | | | p53 | | | |
|---|---|---|---|---|---|---|---|---|
| | *n* | Median ng mg$^{-1}$ proteins | Mean ng mg$^{-1}$ proteins | 20–80th percentile | *n* | Median ng mg$^{-1}$ proteins | Mean ng mg$^{-1}$ proteins | 20–80th percentile |
| 1 | 499 | 0.38 | 0.53 | 0.16–0.85 | 499 | 0.19 | 1.35 | 0.05–0.52 |
| 2 | 222 | 0.21 | 0.30 | 0.10–0.47 | 223 | 0.21 | 0.79 | 0.02–0.48 |
| 3 | 161 | 0.45 | 0.64 | 0.17–0.94 | 161 | 0.15 | 2.85 | 0.05–0.48 |
| 4 | 119 | 0.22 | 0.28 | 0.08–0.43 | 109 | 0.25 | 1.34 | 0.09–0.56 |
| 5 | 93 | 0.24 | 0.31 | 0.11–0.42 | 93 | 0.20 | 1.05 | 0–0.7 |
| 6 | – | ND | ND | ND | 92 | 0.36 | 1.75 | 0.13–0.78 |
| 7 | – | ND | ND | ND | 54 | 0.18 | 1.05 | 0.09–0.42 |
| Total | 1094 | 0.31 | 0.46 | 0.13–0.72 | 1231 | 0.20 | 1.44 | 0.05–0.54 |

ND: not determined, n: number, uPA: urokinase-type plasminogen activator, p53: p53 protein.

Sangtec Diagnostica, Bromma, Sweden) based on a monoclonal antibody (mAb) two-site single-incubation procedure according to the manufacturer's instructions. Antibody-coated polystyrene tubes serve as the solid phase. For p53, the tubes are coated with Pab 1801 mAb and the isoluminol derivative is conjugated to the mAb DO1. Both mAbs bind to denaturation-resistant epitopes at the N-terminus of the wild-type and mutant p53 protein (Banks et al, 1986; Vojtesek et al, 1992). In the case of uPA, the mAb detects the protein in proenzyme form, the active two-chain uPA, uPA bound to its receptor, and uPA bound to plasminogen activator inhibitor type 1 (PAI-1). The uPA and p53 assay procedures have been described elsewhere (Borg et al, 1995; Fernö et al, 1996). The sensitivity of the LIA was < 0.010 ng ml$^{-1}$ for p53 and < 0.005 ng ml$^{-1}$ for uPA.

### Statistical methods

Comparison between uPA and p53 levels and clinical and histological features were performed by using non-parametric tests. We used the Spearman rank correlation when variables were continuous and the Kruskall–Wallis tests for categorized variables. Two-sided $P$-values below 0.05 were considered significant. Disease-related deaths were scored as events with censoring of the other patients for death due to causes other than disease progression, at the time of last follow-up. Primary failure was defined as the occurrence of a loco-regional and/or distant recurrence. Local recurrence was defined as a tumour arising in the treated breast or as a nodal recurrence. Primary failures were scored as an event, censoring the other patients at the time of the last follow-up or death. Disease-specific survival, disease-free interval and distant recurrence-free interval were calculated from the date of first treatment. Disease-specific survival was the time interval between the date of first treatment and the date of disease-related death. Disease-free interval and distant recurrence-free interval were the time intervals between the date of first treatment and the date of primary failure or distant recurrence respectively. Survival curves were derived from Kaplan–Meier (Kaplan and Meier, 1958) estimates, and survival rates are presented with their standard deviation. The problem of using the so-called 'optimal' cut-points, which could overestimate the effect of uPA and p53, led us to use pre-specified cut-points when examining the functional relationship between these markers and outcome (Altman, 1991; Altman et al, 1994). We considered four prespecified cut-points corresponding to the 20th, 40th, 60th and 80th percentiles in the overall population. In univariate analysis, the influence of uPA and p53 was determined by using the log-rank test (Mantel, 1966). The influence of uPA and p53 on outcome, adjusted for the other prognostic factors, was assessed in multivariate analyses by using the Cox proportional hazards regression model (Cox, 1972) in a forward step-wise procedure. To take account of differences among centres, we stratified the multivariate analyses according to the centre.

As the true relationship between the markers and outcomes could be smooth rather than discontinuous, we compared the fit of the model for the two markers as categorized variables (dummy variables) and continuous variables (with a linear function). In this latter case, considering that the distributions of uPA and p53 are highly positively skewed, the markers were log transformed and compared by using Aikaike's criterion, which gives the best-fit model as that with the minimum Akaike information criterion (Akaike, 1974), allowing comparisons between non-nested models. To investigate whether uPA and p53 are important prognostic factors in node-negative breast carcinoma, we applied a Cox model to this low-risk subgroup. Confounding variables with k subgroups were coded to k-1 dummy variables, yielding a non-linear relation between two subsequent subgroups when $k > 2$. To reveal a time-varying effect of uPA and p53, we tested for a non-proportional effect based on the Schoenfeld residuals (Therneau et al, 1990). Interactions between the prognostic value of uPA and p53 and therapeutic factors were also tested in the Cox model. All these analyses were performed with the S-Plus software package (S-Plus, 1993).

### RESULTS

### uPA and p53

The median uPA level in the 1094 tumours analysed was 0.31 ng mg$^{-1}$ protein (mean 0.46, percentiles 0.13, 0.24, 0.39 and 0.72). The median p53 level in the 1231 tumours analysed was 0.20 ng mg$^{-1}$ protein (mean 1.44, percentiles 0.05, 0.14, 0.27 and 0.54). Table 2 summarizes uPA and p53 values in each participating centre and Table 3 gives the correlations with clinical and histological characteristics. Higher levels of uPA were related to older age, larger tumour size, higher clinical stage, higher

 © Cancer Research Campaign 1999

**Table 3** Correlation between uPA, p53 and clinical or histological characteristics

| Patient | uPA Median | *P*-value | p53 Median | *P*-value |
|---|---|---|---|---|
| Age (years) | | | | |
| ≤ 45 | 0.30 | | 0.2 | |
| 46–65 | 0.30 | 0.05 | 0.18 | 0.01 |
| >65 | 0.35 | | 0.24 | |
| Hormonal status | | | | |
| Premenopausal | 0.31 | | 0.19 | |
| Post-menopausal | 0.31 | 0.89 | 0.20 | 0.12 |
| UICC stage | | | | |
| 0–I | 0.25 | | 0.15 | |
| II | 0.32 | 0.0001 | 0.21 | 0.0007 |
| III–IV | 0.45 | | 0.21 | |
| Pathological tumour size (pT) | | | | |
| ≤ 20 mm | 0.28 | | 0.16 | |
| 21–50 mm | 0.34 | 0.0005 | 0.21 | 0.0001 |
| >50 mm | 0.42 | | 0.24 | |
| Histology | | | | |
| Ductal and other | 0.34 | | 0.21 | |
| Lobular | 0.13 | 0.0001 | 0.10 | 0.0001 |
| Histological grading (SBR) | | | | |
| I | 0.28 | | 0.12 | |
| II | 0.31 | 0.0001 | 0.20 | 0.0001 |
| III | 0.45 | | 0.28 | |
| Axillary lymph node (pN) | | | | |
| None | 0.32 | | 0.19 | |
| 1–3 | 0.32 | 0.33 | 0.21 | 0.73 |
| ≥4 | 0.33 | | 0.20 | |
| ER status | | | | |
| Negative | 0.41 | | 0.18 | |
| Positive | 0.29 | 0.0001 | 0.20 | 0.14 |
| PR status | | | | |
| Negative | 0.33 | | 0.18 | |
| Positive | 0.30 | 0.07 | 0.20 | 0.36 |

ER: Estrogen receptor, PR: progesterone receptor.

histological grade, ductal histology and ER negativity. Higher levels of p53 were related to older age, larger tumour size, higher histological and clinical grade, and ductal histology. Levels of uPA correlated positively with p53 levels ($r = 0.27$, $P < 0.0001$).

### Patient outcome

The median follow-up was 75 months (range 3–197). At the time of the analysis, 109 local-regional recurrences, 281 metastases and 235 deaths (223 disease-related) had been recorded. Disease-specific survival was 86% (±1.0) at 60 months and 76% (±1.2) at 96 months. Primary failure had not occurred in 80% (±1.1) of the patients at 60 months and 64% (±1.5) at 96 months. A total of 80% (±1.1) of the patients were distant recurrence-free at 60 months, compared to 71% (±1.5) at 96 months.

### Univariate analysis

The probabilities of disease-specific survival, disease-free interval and distant recurrence-free interval for the four percentiles are illustrated in Figure 1 for uPA and Figure 2 for p53. High levels of uPA and p53 were significantly associated with shorter disease-specific survival, disease-free interval and distant recurrence-free interval.

**Table 4** Five years results according to uPA and p53

| | Disease-specific survival (%) 5 years (s.d.) | Disease-free interval (%) 5 years (s.d.) | Distant recurrence-free interval (%) 5 years (s.d.) |
|---|---|---|---|
| uPA[a] | | | |
| <0.13 | 95.5 (1.5) | 85.6 (2.5) | 88.0 (2.3) |
| 0.13, 0.24 | 88.5 (2.2) | 78.9 (2.8) | 83.3 (2.6) |
| 0.24, 0.39 | 85.6 (2.5) | 75.8 (3.0) | 79.9 (2.8) |
| 0.39, 0.72 | 84.6 (2.5) | 74.5 (2.5) | 77.8 (2.9) |
| >0.72 | 77.5 (3.0) | 63.6 (3.4) | 68.5 (3.3) |
| | $P < 0.00001$ | $P < 0.00001$ | $P < 0.00001$ |
| p53[a] | | | |
| <0.05 | 91.0 (1.9) | 82.8 (2.4) | 86.4 (2.2) |
| 0.05, 0.14 | 86.1 (2.4) | 79.3 (2.7) | 81.9 (2.6) |
| 0.14, 0.27 | 87.0 (2.3) | 75.5 (2.9) | 80.9 (2.6) |
| 0.27, 0.54 | 90.2 (2.0) | 74.1 (3.0) | 78.6 (2.8) |
| >0.54 | 77.6 (2.8) | 67.5 (3.2) | 71.3 (3.1) |
| | $P = 0.004$ | $P = 0.01$ | $P = 0.005$ |

uPA: urokinase-type plasminogen activator, p53: p53 protein, s.d.: standard deviation, [a]prespecified percentiles.

The 5-year disease-specific survival rates were 95.5% (± 1.5) in patients with uPA values below the 20th percentile, and 77.5% (± 3.0) in those with values above the 80th percentile (Table 4). The 5-year disease-specific survival rate was 91.0% (± 1.9) among patients with p53 values below the 20th percentile, and 77.6% (± 2.8) in those with values above the 80th percentile.

At five years, 85.6% (± 2.5) of patients with uPA values below the 20th percentile, and 63.6% (± 3.4) of those with values above the 80th percentile were free from disease recurrence. At five years, 82.8% (± 2.4) of patients with p53 values below the 20th percentile, and 67.5% (± 3.2) of those with values above the 80th percentile were free of disease recurrence.

At five years, 88.0% (± 2.3) of patients with uPA values below the 20th percentile and 68.5% (± 3.3) of those with values above the 80th percentile were free of distant recurrence. At five years, 86.4% (± 2.2) of patients with p53 values below the 20th percentile and 71.3% (± 3.1) of those with values above the 80th percentile were free of distant recurrence.

### Multivariate analysis – overall population

Besides p53 and uPA, the following variables were assessed in multivariate analysis: age at first treatment, menopausal status, clinical stage, lymph node involvement, histoprognostic grading and steroid receptor status. Table 5 shows the results of the final Cox models. The risk of disease-related death increased with positive lymph nodes, high uPA levels, SBR III histological grade, ER-negative tumours and a tumour size larger than 20 mm. The disease-free interval was shorter in case of high uPA levels, more than three invaded lymph nodes, SBR II and III histological grade, tumour larger than 20 mm and ER-negative tumours. The risk of developing distant recurrence increased with more than three invaded lymph nodes, high uPA levels, tumour size larger than 20 mm, SBR II and III histological grade, and ER-negative tumours.

For these three outcomes, p53 status (categorized or continuous) was not an independent prognostic factor, whether or not uPA was included in the model (not shown here). According to the Akaike criterion, the best fit was obtained when uPA was included

© Cancer Research Campaign 1999 

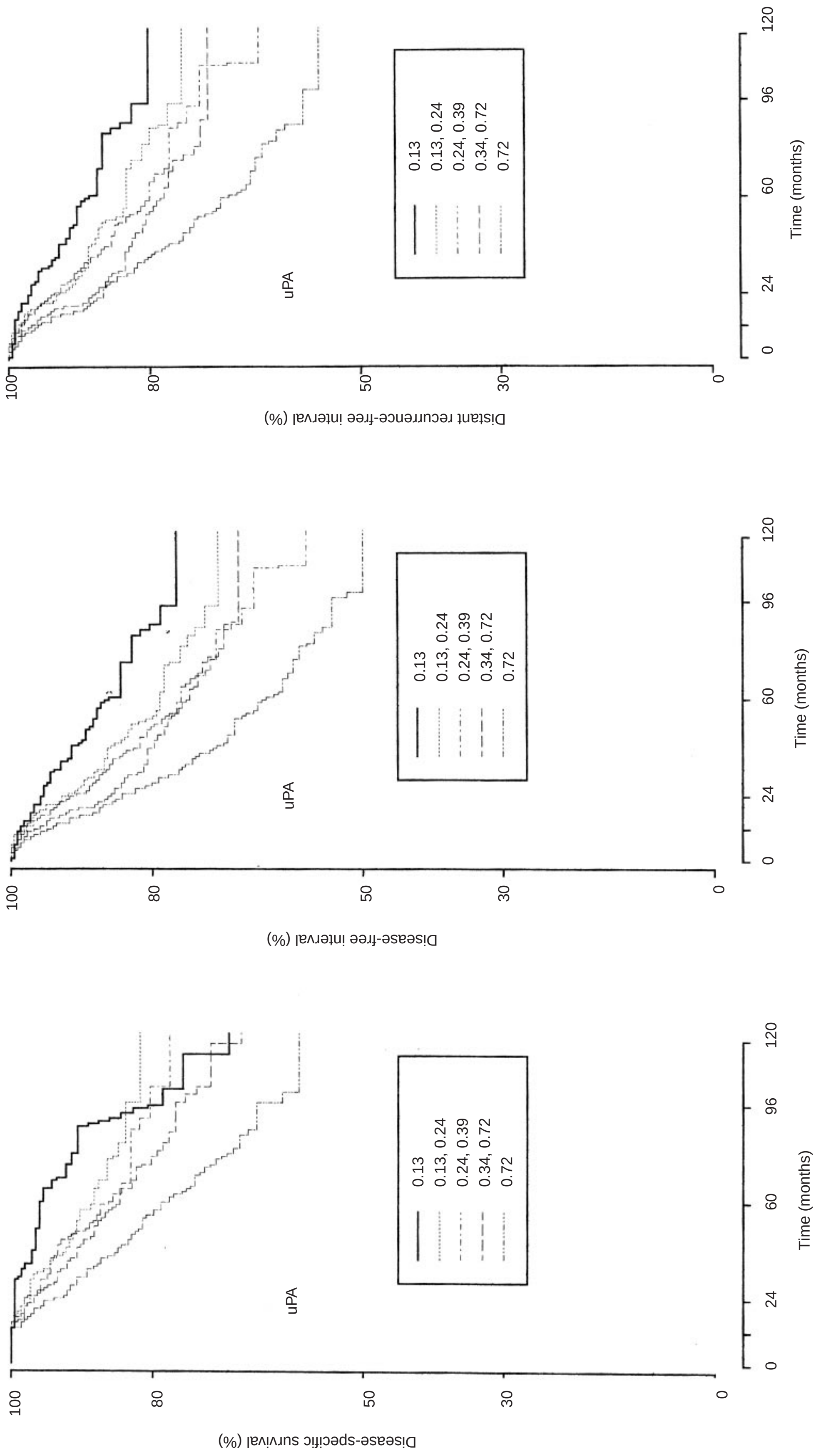

**Figure 1** Disease-specific, disease-free and distant recurrence-free survival curves according to uPA quantiles

 *© Cancer Research Campaign 1999*

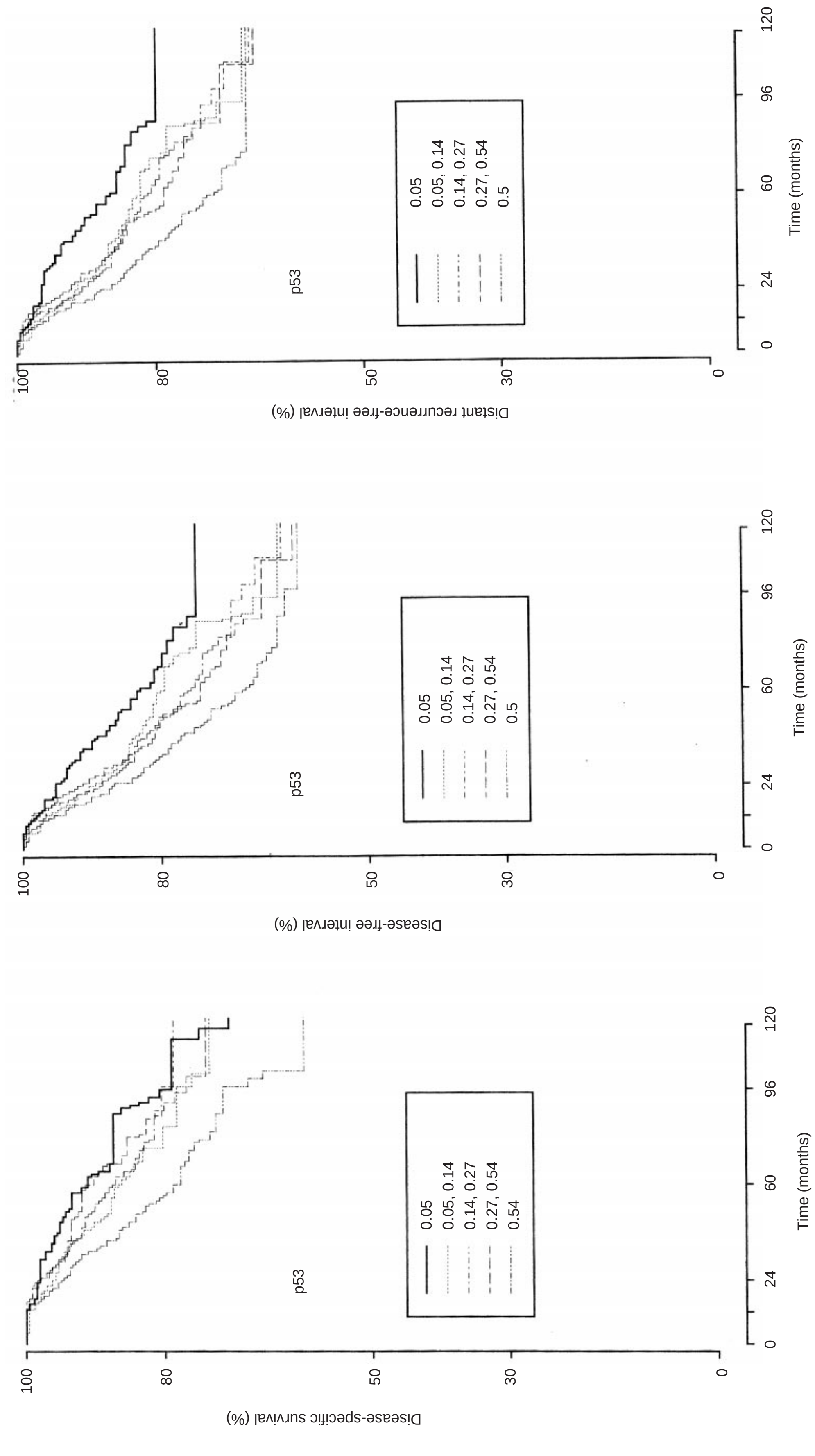

**Figure 2** Disease-specific, disease-free and distant recurrence-free survival curves according to p53 quantiles

**Table 5** Multivariate analysis (overall population)

| Variable | e | Disease-specific survival[a] | e | Disease-free interval[a] | e | Distant-recurrence-free interval[a] |
|---|---|---|---|---|---|---|
| Axillary node status (pN) | 1 | | 2 | | 1 | |
| None | | 1 | | 1 | | 1 |
| 1–3 | | 1.66 [1.14–2.42] | | 1.16 [0.83–1.62][d] | | 1.31 [0.91–1.88][d] |
| >3 | | 3.69 [1.76–5.74] | | 2.29 [1.63–3.22] | | 2.69 [1.88–3.86] |
| uPA | 2 | | 1 | | 2 | |
| (log) | | 1.50 [1.26–1.79] | | 1.45 [1.23–1.69] | | 1.39 [1.19–1.62] |
| Histological grading (SBR) | 3 | | 3 | | 4 | |
| I | | 1 | | 1 | | 1 |
| II | | 1.90 [0.91–4.01][d] | | 2.22 [1.26–3.91] | | 2.10 [1.15–3.83] |
| III | | 2.55 [1.20–5.47] | | 2.88 [1.88–4.40][c] | | 2.50 [1.03–5.30][c] |
| ER | 4 | | 5 | | 5 | |
| Positive | | 1 | | 1 | | 1 |
| Negative | | 1.76 [1.26–2.45] | | 1.43 [1.09–1.88] | | 1.40 [1.05–1.87] |
| Pathological tumour size (pT) | 5 | | 4 | | 3 | |
| ≤ 20 mm | | 1 | | 1 | | 1 |
| > 20 mm | | 1.58 [1.06–2.35] | | 1.84 [1.34–2.55] | | 1.85 [1.30–2.62] |
| p53 | | | | | | |
| (log) | | 1.14 [0.82–1.59][b] | | 0.98 [0.90–1.06][b] | | 1.14 [0.85–1.54][b] |

uPA: urokinase-type plasminogen activator as continuous variable (log transformed), p53: p53 protein as continuous variable (log transformed). [a]Relative risk and confidence interval 95% (relative risk are referred to the best prognosis group) [b]non-significant, [c]non-significant with the upper group, [d]non-significant with the lower group, e: step entry in the Cox model.

as a continuous variable. The risk of death, disease recurrence and distant recurrence increased by 1.50, 1.45 and 1.39 times, respectively, when log (uPA) increased by one unit. For instance, a patient with a uPA value of 0.72 ng mg$^{-1}$ protein (the 80th percentile) was 1.5 times more likely to die than a patient with a uPA value of 0.26 ng mg$^{-1}$ of protein (above the 40th percentile). The only parameter for which a significant time-varying effect was observed was ER status ($P < 0.001$). No time-dependent effect was observed for uPA, whatever the outcome. When testing interaction terms in the model, the effect of uPA and p53 was of the same magnitude in the different treatment groups (hormone therapy, chemotherapy, radiotherapy).

### Multivariate analysis – node-negative patients (Table 6)

Patients with high uPA levels, under 45 years old and with a histological grade higher than I had a shorter disease-free interval and an increased risk of distant recurrences. Except for age, the same factors were associated with an increased risk of disease-related death. The risk of death, disease recurrence and distant recurrence increased by 1.96, 1.63 and 1.85 times, respectively, when log (uPA) increased by one unit. p53 had no significant influence on any of the three outcomes.

**Table 6** Multivariate analysis (node-negative patients)

| Variable | e | Disease-specific survival[a] | e | Disease-free interval[a] | e | Distant-recurrence-free interval[a] |
|---|---|---|---|---|---|---|
| uPA (log) | 1 | | 1 | | 1 | |
| | | 1.96 [1.42–2.71] | | 1.63 [1.31–2.02] | | 1.85 [1.41–2.42] |
| Histological grading (SBR) | 2 | | 3 | | 3 | |
| I | | 1 | | 1 | | 1 |
| II | | 2.67 [1.06–6.75] | | 2.78 [1.43–5.42] | | 2.67 [1.27–5.59] |
| III | | 2.91 [1.36–8.16][c] | | 2.72 [1.20–5.30][c] | | 2.57 [1.30–5.80][c] |
| Age | | | 2 | | 2 | |
| ≤ 45 years | | 1.21 [0.67–2.17][b] | | 1.86 [1.24–2.79] | | 1.75 [1.10–2.77] |
| > 45 years | | 1 | | 1 | | 1 |
| p53 | | | | | | |
| (log) | | 0.94 [0.81–1.09][b] | | 0.96 [0.85–1.07][b] | | 0.98 [0.86–1.11][b] |

uPA: urokinase-type plasminogen activator as continuous variable (log transformed), p53: p53 protein as continuous variable (log transformed). [a]Relative risk and confidence interval 95% (relative risk are referred to the best prognosis group) [b]non-significant, [c]non-significant with the upper group, [d]non-significant with the lower group, e: step entry in the Cox model.

 *© Cancer Research Campaign 1999*

### DISCUSSION

In this study, uPA and p53 were detected with a reliable LIA quantitative assay, which can easily be performed on cytosols prepared for steroid receptor analysis. This biochemical method has the advantage of being automated, reproducible, simple and adaptable to routine clinical use.

This was a multicentre study with inherent advantages and pitfalls. Among the advantages is the large number of patients, meaning that valid conclusions can be drawn despite a heterogeneous population. The variations in uPA and p53 values from one centre to another were probably not due to differences in the way the assay was carried out. Indeed, as part of the quality control process, identical specimens were assayed in several centres and the results were not significantly different from one centre to another. Moreover, an automatic method was used, minimizing inter-laboratory variations. However, there were differences among the centres in the storage of tumours or cytosols, and these probably led to differences in uPA and, to a lesser degree, p53 distribution. Higher uPA values were observed in centres which specifically prepared cytosols for this study. Differences could also be ascribed to regional dissimilarities in patient selection. The multivariate statistical analysis took this centre effect into account and the results were adjusted for it.

Comparisons of the results of different published studies on the prognostic value of uPA and p53 are difficult, mainly because different cut-points were used (Table 7). UPA and p53 values and, consequently, the cut-points, are highly dependent on sample storage conditions, assay methodology, the population selected and statistical methods. Regarding this latter point, continuous variables are frequently analysed using the so-called ‘optimal cutpoint’ approach that is known to overestimate the effect of variables (Altman et al, 1994) and rule out comparisons between studies. In this large series, with a view to later comparisons with other studies, we chose to investigate the prognostic value of uPA and p53 by using four prespecified percentiles based on the overall population. This allowed us to analyse the relationship between the markers and outcomes. The linear relationship in our final model is in adequacy with the results of the univariate survival analysis.

Published studies generally show a correlation between p53 protein accumulation or point mutations of the p53 gene and an aggressive tumour phenotype (Veculescu and El-Deiry, 1996). Immunohistochemistry is the most widely used method, with different antibodies and different definitions of tumour positivity, leading to different results regarding prognostic or predictive value (Allred et al, 1993; MacGrogan et al, 1995). In contrast, there have been only a few reports on p53 gene alteration in large series of breast cancer (De Witte et al, 1996; Sjörgen et al, 1996; Falette et al, 1998).

We measured p53 with a quantitative immunoluminometric method (LIA) with antibodies 1801 and DO1, as described by Borg et al (1995) and, later, De Witte et al (1996), Berns et al (1997) and Peyrat et al (1998). The positive correlations we observed between p53 levels and tumour grade and size are in keeping with the literature. Like Borg et al (1995), De Witte et al (1996), Berns et al (1997) and Peyrat et al (1998), we found that p53 overexpression was associated with shorter distant recurrence-free survival in univariate analysis. The influence of p53 remained significant in the multivariate analyses done by Borg et al (1995) and De Witte et al (1996), while, like Peyrat et al (1998), we found that p53 was no longer significant in a multivariate analysis including uPA and SBR grade. Other authors did not include histological grade or uPA levels in their multivariate studies, and the strong correlation between high grade and p53 positivity may partly explain why Cox analysis including the two parameters reduces the influence of p53. The use of optimum cut-points would also have considerably overestimated the effect of p53 at the expense of other important variables, and this could explain why p53, expressed as a quantitative variable, was not significant in our multivariate analysis. Moreover, we found no interaction between p53 and the type of treatment. As shown in Table 7, the p53 median values in the LIA method are close from one study to another, except for Borg et al (1995) who used cytosols which had been frozen and thawed twice before LIA assay; however, the cut-points and statistical methods used to determine them were quite different as in immunohistochemical studies. The anti-p53 antibodies used in LIA and immunohistochemistry (IHC) assays are specific for denaturation-resistant epitopes of the NH2-terminus of the protein, thus recognizing both mutant and wild-type proteins; in addition, the wild-type protein can be more stable in the absence of p53 gene alterations (Veculescu et al, 1996). Several investigators have found discrepancies between p53 protein expression and mutation status, with large numbers of false-positive and false-negative results (Elledge et al, 1994; Sjögren et al, 1996; Falette et al, 1998); negative results cannot rule out mutations, and

**Table 7** Different cut-off values in multivariate analysis for uPA and p53 assayed by using luminometric assay

| Study | *n* | Median ng mg$^{-1}$ protein | Cut-point ng mg$^{-1}$ protein | % (+)[a] | Statistical method[b] |
|---|---|---|---|---|---|
| uPA | | | | | |
| Fernö et al, 1996 | 688 | 0.40 | 0.62 | 30% | Minimum *P*-value of the log-rank |
| Bouchet et al, 1998 | 499 | 0.38 | 1.14 | 10% | Minimum *P*-value of the log-rank (with correction) |
| Peyrat et al, 1998 | 634 | 0.30 | 0.5 | 27.5% | Optimum cutoff |
| p53 | | | | | |
| Borg et al, 1995 | 205 | 0.05 | 0.15 | 30% | Minimum *P*-value of the log-rank |
| De Witte et al, 1996 | 142 | 0.40 | 2.5 | 28% | Isotonic regression |
| Berns et al, 1998 | 1491 | 0.20 | 0.38 | 34% | Isotonic regression |
| Peyrat et al, 1998 | 634 | 0.38 | 4 | 13.7% | Optimum cutoff |

*n*: Number included in the study. [a]% of patients uPA or p53 positive according to the chosen cutpoint, [b]Statistical methodology used for analysis.

© Cancer Research Campaign 1999 

positive results must be confirmed by sequencing. De Witte et al (1996), comparing LIA and single-strand conformation polymorphism, found a disagreement in 22% of the 142 cases analysed. Finally, interpretation of p53 protein accumulation in a tumour is difficult whatever the method (LIA or IHC), and prognostic data obtained by gene sequencing are certainly more reliable. The value of IHC and luminometric assays could be restricted to screening for p53 mutations. Moreover, the contribution of a true quantitative method appears limited in the case of p53, the main question being the significance of p53 protein accumulation (Elledge, 1996).

Studies of many different human cancers, including breast cancer, have shown that high levels of uPA correlate with poor outcome. uPA was always assayed biochemically and appears to be one of the strongest prognostic markers so far described in breast cancer as informative as nodal status, in spite of differences in extraction buffers, uPA standards and antibodies, and a lack of uniform cut-points (Duffy, 1996). In this study we used hormone receptor buffer, which yields lower uPA values than Triton X-100 buffer (Janicke et al, 1994*a*; Knoop et al, 1998). However, significant prognostic value has also been observed with cytosols prepared for hormone receptor assays (Foekens et al, 1992; Bouchet et al, 1994, 1998; Jänicke et al, 1994*a*; Fernö et al, 1996; Peyrat et al, 1998). Our technique has the advantage of being automated. Bouchet (1998) recently showed a good concordance between the results of the most widely used enzyme-linked immunosorbent assay (ELISA) test for uPA (American Diagnostica) and the LIA assay, although the two methods were not interchangeable: indeed, lower uPA levels were obtained with luminometry on cytosols prepared for hormone receptor assays. If we compare our results to those of studies using the same assay (Fernö et al, 1996; Peyrat et al, 1998), median uPA values were close (Table 7). We confirm the negative correlation between uPA levels and ER status, and the positive correlations between uPA levels, histological grade and tumour size (Duffy, 1996). In our study, the prognostic value of uPA was comparable to that of lymph node involvement, and a high uPA level was still an independent prognostic indicator in multivariate analysis, confirming previous studies based on ELISA methods (Foekens et al, 1992; Jänicke et al, 1993; Duffy, 1996; Bouchet et al, 1998) or LIA (Fernö et al, 1996; Bouchet et al, 1998; Peyrat et al, 1998).

The search for additional markers in node-negative patients is a major issue. In our analysis, after adjustment for other factors, uPA was the main prognostic factor for the three outcomes in the multivariate analysis of this low-risk group, in agreement with previous data based on ELISA methods (Foekens et al, 1992; Jänicke et al, 1993), LIA (Fernö et al, 1996; Peyrat et al, 1998) or both (Bouchet et al, 1998). uPA is only part of the plasminogen activation system, which includes complex interactions between uPA, its inhibitors PAI-1 and PAI-2, and its receptor uPAR, in different cell components. A study by Schmitt et al (1997) suggested that the estimated hazards ratio for uPA could fall with time, contrary to PAI-1, which remained stable, but the presented figures did not show clearly a non-proportional hazards effect for uPA. We found no time-related fall in the hazards ratio for uPA, whereas this effect was highly significant for ER status, in accordance with Pichon et al (1996). PAI-1 has also been found more predictive than uPA, particularly in node-negative patients (Jänicke et al, 1993; Bouchet et al, 1994; Foekens et al, 1994), but its pathophysiological role is unclear, contrary to uPA (Andreasen et al, 1997). The importance of uPA as a prognostic factor in breast cancer is underlined by its role in metastasis, and by its value in node-negative patients, in whom uPA is not redundant with proliferative markers, including SBR grade. We found no interaction between uPA and treatment modalities. However, it is important to take uPA into account in randomized prospective trials as a new treatment stratification parameter for node-negative patients. A question of special interest is whether high uPA levels, which place node-negative patients in a high-risk group, affect the response to therapy, and this issue is currently being addressed in Jänicke's clinical trial (1994*b*).

In conclusion, this large multicentre study confirms the importance of uPA in predicting the outcome of node-negative breast cancer patients, without using optimal cut-points and in spite of differences among centres (taken into account in the multivariate analyses). The predictive power of uPA in these conditions argues for general use of this variable in clinical practice. In our series, however, the quantitative assessment of p53 protein accumulation added no prognostic information in the multivariate approach including uPA and histological grade.

### ACKNOWLEDGEMENTS

We thank all the pathologists and clinician teams of the different centres who actively participated in the study. We are also grateful for the expert technical assistance of the persons involved in the luminometric assays of uPA and p53 in each centre. The help of Sangtec in the uPA and p53 luminometric assays is gratefully acknowledged.

### REFERENCES

Akaike H (1974) A new look at the statistical model identification. *IEEE Transactions on Automatic Control* **19**: 716–723

Allred DC, Clark GM, Elledge R, Fuqua SA, Brown RW, Chamness GC, Osborne CK and McGuire WL (1993) Association of p53 protein expression with tumor cell proliferation rate and clinical outcome in node-negative breast cancer. *J Natl Cancer Inst* **85**: 200–206

Altman DG (1991) Categorising continuous variables. *Br J Cancer* **64**: 975

Altman DG, Lausen B, Sauerbrei W and Schumacher M (1994) Dangers of using 'optimal' cutpoints in the evaluation of prognostic factors. *J Natl Cancer Inst* **86**: 829–835

Andreasen PA, Kioller L, Christensen L and Duffy MJ (1997) The urokinase-type plasminogen activator system in cancer metastasis: a review. *Int J Cancer* **72**: 1–22

Banks L, Matlashewski G and Crawford L (1986) Isolation of human p53 specific monoclonal antibodies and their use in the studies of human p53 expression. *Eur J Biochem* **159**: 529–534

Berns EMJJ, De Witte HH, Klinj JGM, Willman K, Look MP, Meijer-van Gelder ME, Benraad T and Foekens J (1997) Prognostic value of TP53 protein accumulation in human primary breast cancer: an analysis by luminometric immunoassay on 1491 tumor cytosols. *Anticancer Res* **17**: 3003–3006

Bloom HJ and Richardson WW (1957) Histological grading and prognosis in breast cancer. *Br J Cancer* **11**: 359–377

Borg A, Lennersand J, Stenmark-Askmalm M, Fernö M, Brisfors A, Öhrvik A Stål O, Killander D, Lane D and Brundell J (1995) Prognostic significance of p53 overexpression in primary breast cancer; a novel luminometric immunoassay applicable on steroid receptor cytosols. *Br J Cancer* **71**: 1013–1017

Bouchet C, Spyratos F, Martin PM, Hacène K, Gentile A and Oglobine J (1994) Prognostic value of urokinase activator and plasminogen activator inhibitors PAI-1 and PAI-2 in breast carcinoma. *Br J Cancer* **69**: 398–405

Bouchet C, Spyratos F, Hacène K, Durcos L, Bécette V and Oglobine J (1998) Prognostic value of urokinase activator in primary breast carcinoma: comparison of two immunoassay methods. *Br J Cancer* **77**: 1495–1501

Cox DR (1972) Regression models and life tables (with discussion). *J R Stat Soc B* **34**: 187–202

De Witte HH, Foekens JA, Lennerstrand J, Smid M, Look MP, Klijn GM, Benraad TJ and Berns MJJ (1996) Prognostic significance of tp53 accumulation in

 © Cancer Research Campaign 1999

human primary breast cancer: comparison between a rapid quantitative immunoassay and SSCP analysis. *Int J Cancer* **69**: 125–130

Duffy MJ (1996) Proteases as prognostic markers in cancer. *Clin Cancer Res* **2**: 613–618

Elledge RM (1996) Assessing p53 status in breast cancer prognosis; where should you put the thermometer if you think your p53 is sick? *J Natl Cancer Inst* **88**: 141–142

Elledge RM, Clark GM, Fuqua SAW and Yu YY (1994) Allred C: p53 protein accumulation detected by five different antibodies: relationship to prognosis and heat shock protein 70 in breast cancer. *Cancer Res* **54**: 3752–3757

EORTC (European Organization for Research and Treatment of Cancer) (1980) Breast cancer cooperative group. Revision of the standards for the assessment of hormone receptors in human breast cancer. *Eur J Cancer* **16**: 1513–1515

Falette N, Paperin Mp, Treilleux I, Gratadour AC, Peloux N, Mignote H, Tooke N, Lofman E, Iganas M, Brémond A, Ozturk M and Puisieux A (1998) Prognostic value of p53 gene mutations in a large series of node-negative breast cancer patients. *Cancer Res* **58**: 1451–1456

Fernö M, Bendahl, P.O, Borg A, Brundell J, Hirschberg L, Olsson H and Killander D (1996) Urokinase plasminogen activator, a strong independent prognostic factor in breast cancer, analysed in steroid receptor cytosols with luminometric immunoassay. *Eur J Cancer* **5**: 793–801

Figueroa JA, Yee D and McGuire WL (1993) Prognostic indicators in early breast cancer. *Am J Med Sci* **305**: 176–182

Foekens JA, Schmitt M, Van Putten WLJ, Peters HA, Bontenbal M, Jänicke F and Klijn JGM (1992) Prognostic value of urokinase-type plasminogen activator in 671 primary breast cancer. *Cancer Res* **52**: 6101–6105

Foekens JA, Schmitt M, Van Putten WLJ, Peters HA, Kramer MD, Jäunicke F and Klijn JGM (1994) Plasminogen activator inhibitor-1 and prognosis in primary breast cancer. *J Clin Oncol* **12**: 1648–1658

Gottlieb TM and Oren M (1996) p53 in growth control and neoplasia. *Biochem Biophys Acta* **1287**, 77–102

Jänicke F, Schmitt M, Pache L, Harbeck N, Höfler H and Graeff H (1993) Urokinase (uPA) and its inhibitor PAI-1 are strong and independent prognostic factors in node-negative breast cancer. *Breast Cancer Res Treat* **24**: 195–208

Jänicke F, Pache L, Schmitt M, Ulm K, Thomssen C, Prechtl A and Graeff H (1994*a*) Both the cytosols and detergent extracts of breast cancer tissues are suited to evaluate the prognostic impact of the urokinase-type plasminogen activator and its inhibitor plasminogen activator inhibitor-type 1. *Cancer Res* **54**: 2527–2530

Jänicke F, Thomssen C, Pache M, Schmitt M and Graeff H (1994*b*) Urokinase (uPA) and PAI-1 as selection criteria for adjuvant chemotherapy in axillary node-negative breast cancer patients. In *Prospects in Diagnosis and Treatment of Breast Cancer,* Schmitt M, Graeff H and Kendermann G (eds) pp. 207–218. Excerpta Medica: Amsterdam

Kaplan EL and Meier P (1958) Nonparametric estimation from incomplete observations. *J Am Stat Assoc* **53**: 457–481

Knoop A, Andreasen PA, Andreasen JA, Hansen S, Laenkholm AV, Simonsen ACW, Andersen J, Overgaard J and Rose C (1998) Prognostic significance of urokinase-type plasminogen activator and type plasminogen activator inhibitor-1 in primary breast cancer. *Br J Cancer* **77**: 932–940

MacGrogan G, Bonichon F, de Mascarel I, Trojani M, Durand M, Avril A and Coindre JM (1995) Prognostic value of p53 in breast invasive ductal carcinoma: an immunohistochemical study of 942 cases. *Breast Cancer Res Treat* **36**: 71–81

Mantel N (1966) Evaluation of survival data and two rank order statistics arising in its consideration. *Cancer Chemother Res* **50**: 163–170

Peyrat JP, Vanlemmens L, Fournier J, Huet G, Révillon F and Bonneterre J (1998) Prognostic value of p53 and urokinase-type plasminogen activator in node negative human breats cancers. *Clin Cancer Res* **4**: 189–196

Pichon MF, Broët P, Magdelenat H, Delarue JC, Spyratos F, Basuyau JP, Saez S, Rallet A, Courrière R, Millon R and Asselain B (1996) Prognostic value of steroid receptors after long-term follow-up of 2257 operable breast cancer. *Br J Cancer* **73**: 1545–1552

Porter-Jordan K and Lippman ME (1994) Overview of the biologic markers of breast cancer. *Hematol Oncol Clin North Am* **8**: 73–100

Scarff RW and Torloni H (1968) *Histological Typing of Breast Tumors*, pp. 13–20. World Health Organization: Geneva 13–20

Schmitt M, Thomssen C, Ulm K, Selderer A, Harbeck N, Höfler H, Jänicke F and Graeff H (1997) Time-varying prognostic impact of tumour biological factors urokinase (uPA), PAI-1 and steroid hormone receptor status in primary breast cancer. *Br J Cancer* **76**: 306–311

Sjörgren S, Iganas M, Norberg T, Lindgren A, Nordgren H, Holmberg L and Bergh J (1996) The p53 gene in breast cancer: prognostic value of complementary DNA sequencing versus immunohistochemistry. *J Natl Cancer Inst* **88**: 173–182

S-Plus Version 3.2 Guide to Statistics (1993) Statistical Sciences: Seattle, WA

Therneau TM, Grambsch PM and Fleming TR (1990) Martingale-based residuals for survival models. *Biometrika* **77**: 147–160

Thor AD and Yandell DW (1993) Prognostic significance of p53 overexpression in node-negative breast carcinomas: preliminary results support cautious optimism. *J Natl Cancer Inst* **85**: 176–177

UICC (Union Internationale Contre le Cancer) *(1988) TNM Classification des Tumeurs Malignes,* Springer-Verlag: Paris pp. 99–106

Velculescu VE and El-Deiry WS (1996) Biological and clinical importance of the p53 tumor suppressor gene. *Clin Chem* **42**: 858–868

Vojtesek B, Bártek J, Migdley CA and Lane DP (1992) An immunochemical analysis of the human nuclear phosphoprotein p53. New monoclonal antibodies and epitope mapping using recombinant p53. *J Immuno Methods* **151**: 237–244

© Cancer Research Campaign 1999
